# Local and Remote Digital Pre-Distortion for 5G Power Amplifiers with Safe Deep Reinforcement Learning †

**DOI:** 10.3390/s25196102

**Published:** 2025-10-03

**Authors:** Christian Spano, Damiano Badini, Lorenzo Cazzella, Matteo Matteucci

**Affiliations:** 1 Politecnico di Milano, Piazza Leonardo da Vinci 32, 20133 Milan, Italy; lorenzo.cazzella@polimi.it (L.C.); matteo.matteucci@polimi.it (M.M.); 2 Huawei Technologies Italia S.r.l., Via Martesana 12, 20055 Vimodrone, Italy; damiano.badini@huawei.com

**Keywords:** deep reinforcement learning, digital pre-distortion, power amplifier

## Abstract

The demand for higher data rates and energy efficiency in wireless communication systems drives power amplifiers (PAs) into nonlinear operation, causing signal distortions that hinder performance. Digital Pre-Distortion (DPD) addresses these distortions, but existing systems face challenges with complexity, adaptability, and resource limitations. This paper introduces DRL-DPD, a Deep Reinforcement Learning-based solution for DPD that aims to reduce computational burden, improve adaptation to dynamic environments, and minimize resource consumption. To ensure safety and regulatory compliance, we integrate an ad-hoc Safe Reinforcement Learning algorithm, CRE-DDPG (Cautious-Recoverable-Exploration Deep Deterministic Policy Gradient), which prevents ACLR measurements from falling below safety thresholds. Simulations and hardware experiments demonstrate the potential of DRL-DPD with CRE-DDPG to surpass current DPD limitations in both local and remote configurations, paving the way for more efficient communication systems, especially in the context of 5G and beyond.

## 1. Introduction

In mobile wireless communications, cost-effective delivery of high-quality data relies heavily on power amplifiers (PAs), which provide the radio frequency (RF) power essential for communication. The growing demand for higher data rates and extended ranges has led to more powerful and efficient PAs, but this has also intensified challenges related to PA nonlinearities, especially with high Peak-to-Average Power Ratio (PAPR) signals.

High PAPR signals, common in Orthogonal Frequency Division Multiplexing (OFDM), a key 5G technology, have large peaks that strain PAs, reducing efficiency and performance. These nonlinearities, caused by device characteristics, power supply variations, and environmental factors like temperature changes, result in distortion, spectral regrowth, and intermodulation, which degrade signal quality, increase error rates, and cause adjacent channel interference. Mitigating these effects is crucial for ensuring reliable, standards-compliant communication systems.

An earlier version of this paper was presented at the 54th EuMC and was published in its Proceedings [1]. With respect to the previous version, we expanded the discussion on feedback line bottlenecks, added results from the simulated remote setting, explained in more detail the proposed safe RL algorithm with the added pseudocode, and revised its overall clarity and readability.

### 1.1. Digital Pre-Distortion and Limitations

To address the nonlinearities of PAs, various linearization techniques have been proposed. Among them, Digital Pre-Distortion (DPD) is the most widely used and effective solution. Real-time DPD implementations further enhance its capabilities by dynamically adapting to changing operating conditions, ensuring consistent performance. However, traditional DPD systems face significant challenges in the feedback path, where updating coefficients requires the entire signal to be sampled by high-bandwidth, power-intensive Analog-to-Digital Converters (ADCs). For instance, in 5G FR2 with 400 MHz bandwidth, conventional DPD systems must capture and process one or more complete OFDM symbols for coefficient estimation. With a subcarrier spacing of 120 kHz, each OFDM symbol lasts for approximately 8.33μs and requires the processing of approximately 66 kbits of IQ data per symbol (assuming 10-bit ADCs). This data must undergo computationally intensive nonlinear processing tasks such as filtering, phase alignment, PA modeling, and matrix inversion, further straining conventional processing approaches. From a hardware complexity perspective, conventional DPD feedback paths require energy-intensive complete receiver chains comprising down-conversion mixers, multi-stage filtering, variable-gain amplifiers, and high-speed ADCs operating at GHz rates, with high-speed ADCs alone consuming tens to hundreds of milliwatts depending on resolution and sampling frequency.

To reduce these burdens, several works have proposed coefficient adaptation mechanisms that rely on feature selection or reduced-signal representations. For instance, Li et al. [2] introduced pretraining-based feature extraction to accelerate coefficient updates; this approach depends on comprehensive offline calibration and may struggle to adapt to unmodeled operating conditions such as thermal drift or hardware aging. Similarly, Mengozzi et al. [3] employed global feature-based learning for active array linearization; the method relies on pretraining and interpolation across beam and PAPR spaces, which can limit robustness when the array or PA behavior deviates from the training manifold. Wang et al. [4] proposed mesh-selecting techniques to reduce computational costs in PA modeling; their performance hinges on carefully chosen sample selection and still requires waveform-level observations during identification. Finally, undersampling-based observation strategies have been investigated to limit ADC requirements [5]; these solutions introduce additional analog front-end complexity and can suffer from aliasing or information loss in wideband FR2 scenarios. While these methods significantly improve efficiency compared to conventional DPD, they still incur non-negligible overhead from pretraining, calibration, or specialized hardware, leaving their complexity and power consumption tightly coupled to bandwidth and observation path requirements.

To tackle these issues, strategies like band-limited digital predistortion (BL-DPD) have been explored. Methods employing BPF [6] or sub-Nyquist sampling [7] aim to handle wideband signals without increasing ADC bandwidth. However, these approaches often lose information vital for bandwidth-hungry applications like 5G. Recent advancements, such as IOBL-DPD [8] architectures, mitigate this information loss by using switching mechanisms to selectively capture PA inband and outband data in each iteration without expanding ADC bandwidth. Although effective in preserving information, these methods introduce additional challenges in complexity and energy consumption. Similar complications arise in remote DPD configurations [9], where the feedback path operates over-the-air (OTA) via an RF link, as seen in point-to-point networks with separate transmitter (TX) and remote terminal (RX) nodes. In such systems, the RF link’s frequency response affects the transmitted signal, and the lack of direct access to the original signal complicates coefficient estimation. Moreover, these setups face challenges related to complexity, latency, and potential errors from extensive data exchange between TX and RX.

### 1.2. Deep RL in Wireless Systems

Deep Reinforcement Learning (DRL) has gained significant attention for its ability to solve complex decision-making problems in dynamic environments. By leveraging trial-and-error learning and reward-driven optimization, DRL has been successfully applied across various domains, including wireless communication systems [10,11].

In particular, in the physical layer, DRL has been employed to enhance adaptability and efficiency in tasks such as channel estimation, coding scheme selection, self-interference cancellation, and power control [12,13,14,15,16]. These tasks share characteristics with DPD, including high-dimensional input spaces, nonlinear system behaviors, and dynamic operating conditions, all of which pose challenges to conventional model-based approaches. Given that DPD operates at the physical layer to linearize PAs under varying signal and hardware conditions, applying DRL to DPD is a logical extension of these prior applications. DRL’s ability to learn optimal control policies in real time without requiring precise system models makes it particularly well-suited for the nonlinear and time-varying nature of PA distortion.

Although relatively few DRL-based DPD works exist in the literature, recent studies such as [17,18] have explored this direction. Wu et al. [17] propose a symbol-based over-the-air RL framework that relies on downsampled symbol observations from the receiver and optimizes a symbol-level loss related to the symbol error rate (SER). While this approach successfully avoids a full-rate ADC and shows SER improvements compared to ILA, it has some limitations: it requires reliable symbol recovery at the receiver, it is evaluated under AWGN with a GMP PA model rather than in wideband FR2 hardware, and it does not directly optimize spectral metrics such as ACLR that are critical for compliance in 5G systems. Zhang et al. [18] take a different approach, applying Deep Q-Learning to automatically search the structure of GMP models, explicitly balancing NMSE/ACPR performance against model complexity. Their Auto-GMP achieves competitive NMSE/ACPR results on a 2.4 GHz, 100 MHz Doherty PA with fewer basis functions, but it fundamentally relies on waveform-level NMSE/ACPR feedback and is best suited as an offline model selection tool rather than for online adaptation under minimal feedback.

In contrast, our proposed DRL-DPD method is explicitly designed for scalar-feedback operation in wideband 5G scenarios. By relying only on performance indicators such as Adjacent Channel Leakage Ratio (ACLR) and Signal-to-Noise Ratio (SNR) and incorporating a safe policy-based learning mechanism, our framework enables efficient online coefficient adaptation while also actively preventing exploration steps that could cause PA overdrive or spectral-mask violations. This built-in safety layer makes our approach more suitable for real hardware deployments, where maintaining regulatory compliance and avoiding device stress must be guaranteed.

Among various DRL algorithms, policy-based methods like Deep Deterministic Policy Gradient (DDPG) [19] are well-suited for continuous control problems, making them an effective choice for optimizing dynamic processes in wireless systems. In this work, we leverage DDPG for our proposed DRL-based DPD framework, enabling adaptive coefficient updates while ensuring efficient learning and control. The computational requirements for DDPG inference are particularly well-matched to DPD applications, as coefficient updates typically occur at much slower timescales (milliseconds to seconds) compared to traditional signal processing operations (microseconds), allowing sufficient time for neural network computations even on modest embedded processors. Furthermore, modern hardware accelerators (e.g., Graphics Processing Units (GPUs) and Tensor Processing Units (TPUs)) tailored for neural network operations enhance the feasibility of deploying DRL-based solutions in real-world communication systems, though such specialized hardware is not strictly necessary for the proposed approach.

### 1.3. Contributions and Outline

In this paper, we propose the following contributions:

- A novel DRL-based DPD method, named DRL-DPD, designed to address local and remote DPD challenges by reducing hardware complexity and energy costs.
- An extensive evaluation of DRL-DPD in both simulated (modeled PA) and real-hardware environments, demonstrating its scalability and adaptability to time-varying PA behavior (e.g., due to nonlinearities, thermal effects, etc.).
- The introduction of CRE-DDPG (Cautious-Recoverable-Exploration DDPG), a Safe-RL algorithm that ensures safety by constraining the agent’s exploration to avoid unacceptable ACLR violations when updating the DPD coefficients.

In the following, Section 2 introduces DRL-DPD, its architectures, and its implementation for both configurations. Next, Section 3 presents results from simulated environments. Lastly, we discuss DRL-DPD’s performance in a local setup on real hardware and introduce a tailored safe-RL algorithm for secure DPD coefficient updates.

## 2. DRL-DPD

This paper aims to minimize feedback-line costs in local and remote DPD without adding further complexity with respect to traditional methods by leveraging the adaptiveness of DRL. To achieve this, we propose DRL-DPD, a novel DPD approach based on DRL that effectively addresses local and remote DPD challenges. Local DPD refers to configurations where the PA and DPD processing unit are co-located, enabling direct access to the PA output for feedback measurements. Remote DPD, instead, operates in distributed scenarios where the PA is physically separated from the DPD controller, typically connected via wireless links, requiring over-the-air feedback mechanisms.

Instead of using the entire output signal bandwidth to update DPD coefficients, DRL-DPD only requires a single scalar measure: the ACLR for local DPD and SNR for remote. These metrics reflect PA-induced nonlinearities and are used by the DRL agent to guide coefficient updates based on experience.

In the local case, ACLR is computed via a narrowband bandpass filter and power detector placed at the PA output. This narrowband approach selectively measures power in targeted spectral sidebands rather than capturing the full signal bandwidth. By controlling these specific sideband components, the overall spectral regrowth is effectively suppressed, thereby improving ACLR and linearizing the PA. This simplified feedback path, comprising a narrowband filter, power detector (diode), and low-speed ADC, consumes negligible power compared to traditional wideband receivers, representing orders of magnitude reduction in both hardware complexity and energy consumption.

In the remote case, direct ACLR computation is impractical due to signal interference from other transmitters. However, the receiver always estimates SNR (directly related to EVM), making it a convenient and readily available feedback signal for the DRL agent. Since improving ACLR or SNR usually benefits the other, each serves as a reliable proxy for nonlinear distortion in its respective context.

### 2.1. Local DRL-DPD

**Figure 1:**
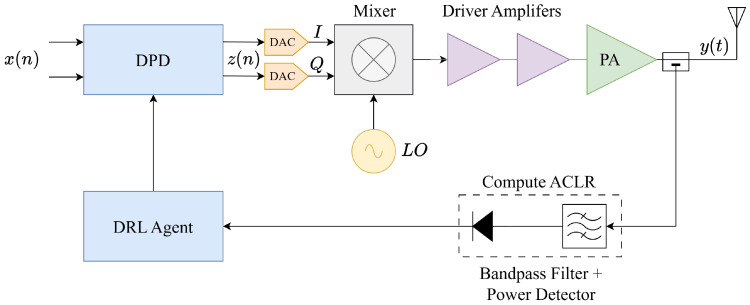
Local DRL-DPD architecture. Complexity and costs are dramatically reduced as we remove the expensive ADC in the feedback line.

An illustration of the proposed architecture in the local scenario is shown in Figure 1. At time *t*, the DPD module receives as input the original signal, x(n), and outputs the pre-distorted signal, z(n), using the current estimated coefficients, $s_{t}$. In particular, the DPD can be designed as usual, that is, by using a nonlinear model able to model the inverse characteristic of the PA (e.g., an MP model [20]). After the DPD, two Digital-to-Analog Converters (DACs) convert the pre-distorted signal that passes through an IQ-Modulator (or mixer). An LO up-converts the frequency from digital Intermediate Frequency (IF) to RF and then driver amplifiers boost the signal level for the PA. The PA takes as input this boosted (pre-distorted) signal and outputs a signal that, ideally, should be a linear amplification of x(n). Subsequently, using a directional coupler, a portion of the PA output power is passed to a narrowband filter and a power detector (i.e., a diode) specifically designed to isolate and measure the power in a targeted portion of the spectral sidebands. This selective measurement captures the out-of-band emissions that directly correlate with PA nonlinearity, enabling effective ACLR estimation without requiring wideband signal capture. Notably, this measurement process does not need to be performed in real-time at the symbol rate as the PA’s characteristics change slowly, especially once in convergence. The inherent group delay introduced by the narrowband filter is negligible in this context, as it operates on timescales orders of magnitude faster than the PA’s characteristic variations, which are primarily driven by slow thermal effects. In turn, the measured ACLR is passed to the DRL agent that, based on the current coefficients and its current knowledge, decides upon the update to assign to the coefficients to improve the metric. After having decided, the coefficients of the DPD model are updated accordingly and the process continues iteratively.

This architecture fundamentally differs from conventional DPD in terms of hardware requirements and associated costs: our narrowband filter and power detector consume significantly less power and require no active amplification or mixing stages. The resulting hardware cost reduction is substantial, as passive filters and simple diode detectors cost a fraction of complete receiver systems.

An important advantage of the local configuration is that signal statistics conditioning remains highly predictable. Since DRL-DPD operates within the transmitter itself, signal characteristics remain stable and controlled throughout the learning process. Any variations in signal statistics that may occur (e.g., due to adaptive power control or modulation scheme changes) are known a priori and controlled by the system, making them easily manageable by the DRL-DPD framework through its inherent adaptive learning capability.

### 2.2. Remote DRL-DPD

**Figure 2:**
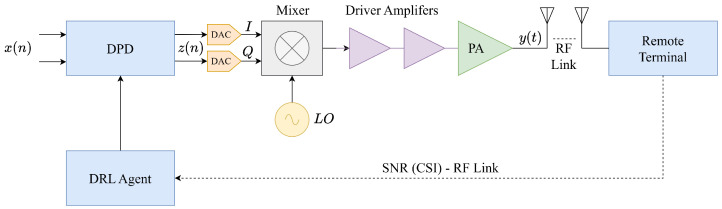
Remote DRL-DPD architecture. Complexity and costs are practically zeroed-out from the remote terminal perspective.

In the context of remote DPD (see Figure 2), we have a transmitter (which comprises the same forward path as the local case) that communicates with a remote terminal via an RF-link. In DRL-DPD, the remote terminal has to only reply to the transmitter as it would have done without any DPD implementation, that is, without further computations. Indeed, according to the 5G communication standard, in this response, there is a measure that is always sent in the CSI, which is the SNR. In other words, SNR estimation is an inherent part of all bidirectional communication standards (e.g., Wi-Fi, 5G), performed routinely by receivers for channel quality assessment, regardless of distance or system configuration. Once the transmitter receives the SNR, this is exploited by the DRL agent to adjust the DPD coefficients.

Although the wireless channel introduces variable signal statistics that could complicate remote DPD operation, this challenge is effectively mitigated in practical deployments. Systems employing remote DPD are typically configured in point-to-point scenarios where channel variations are systematically compensated by the receiver’s equalizer, which is a standard function present in all communication systems. This linear equalizer effectively neutralizes channel-induced distortions, isolating nonlinear transmitter distortions as the primary SNR-limiting factor. Consequently, the SNR feedback directly reflects PA nonlinearity effects, enabling the DRL agent to adapt DPD coefficients based on meaningful distortion information rather than channel-corrupted measurements.

### 2.3. DRL-DPD Implementation

To implement DRL-DPD, we basically need to define the engine that controls everything: the DRL agent. To do that, we need to define the four core elements it is built upon, that is, the *states*, *actions*, *new states,* and *rewards* [21].

In our case, the states correspond to the current complex coefficients of the DPD model, denoted as $s_{t}\in C^{J}$, where *J* corresponds to the total order of the model (e.g., J=K·M for an MP model). In practice, for convenience, we defined $s_{t}\in R^{2J}$, that is, as a real vector of coefficients. In particular, $s_{t}$ is designed such that the first *J* components are the real parts of the coefficients, whilst the remained ones correspond to the imaginary parts. In other terms,

(1) $$s_{t}=\left[\begin{array}{cc} \overset{Real}{\overset{︷}{\begin{array}{ccc} s_{0}^{t} & \ldots & s_{J-1}^{t} \end{array}}} & \overset{Imaginary}{\overset{︷}{\begin{array}{ccc} s_{J}^{t} & \ldots & s_{2J-1}^{t} \end{array}}} \end{array}\right]$$

For instance, using an MP to model the DPD, we would have

(2) $$z_{t}\left(n\right)=\sum_{k=0}^{K-1}\sum_{m=0}^{M-1}{\bar{s}}_{km}^{t}x\left(n-m\right){\left|x\left(n-m\right)\right|}^{k}$$

where, still, $z_{t}\left(n\right)$ is the DPD output, x(n) is the original signal (DPD input), and ${\bar{s}}_{km}^{t}$ are the complex DPD coefficients such that ${\bar{s}}_{km}^{t}=s_{Mk+m}^{t}+js_{J+Mk+m}^{t}$, where $s_{i}^{t}$ is the *i*-th component of $s_{t}$. Often, only odd-order nonlinearities are included in the model. Nothing changes, except a useful reduction of the state dimension to $2\lceil \frac{J}{2}\rceil$.

The actions, meanwhile, correspond to the updates the agent applies to adjust the coefficients. Indeed, we define an action as a vector of real values with the same dimension of the state, that is, $a_{t}\in R^{2J}$. In other terms,

(3) $$a_{t}=\left[\begin{array}{cccccc} a_{0}^{t} & \ldots & a_{J-1}^{t} & a_{J}^{t} & \ldots & a_{2J-1}^{t} \end{array}\right]$$

where $-1\leq a_{i}^{t}\leq 1$, ∀i=0,1,…,2J−1. This way, the actions are bounded, which means bounding the updates as result. Through experiments, we found out that using such a bound provided higher stability during learning, in contrast to letting the updates be unbounded. This is reasonable as it makes the agent more cautious about its decisions.

New states, denoted as $s_{t}\in R^{2J}$, are the result of applying a particular action $a_{t}$ to the previous estimated coefficients, $s_{t-1}$. In our case, this means to update each state coefficient with the corresponding update. Formally, $s_{t}=s_{t-1}+a_{t}$, that is,

$$\begin{array}{cc} s_{t} & =\left[\begin{array}{cccc} s_{0}^{t-1}+a_{0}^{t} & \ldots & s_{J}^{t-1}+a_{J}^{t} & \ldots \end{array}\right]\\ & =\left[\begin{array}{cccc} s_{0}^{t} & \ldots & s_{J}^{t} & \ldots \end{array}\right] \end{array}$$

Finally, the reward depends on the kind of DPD framework we are considering. If we are dealing with a local DPD, then the ACLR will drive the agent decisions; otherwise, in the case of remote DPD, the agent choices will be driven by the SNR. In more detail, we define the reward as follows:

(4) $$\begin{array}{ccc} R_{L}^{t}=-\left|{\bar{P}}_{ACLR}-P_{ACLR}^{t}\right| & \; & R_{R}^{t}=-\frac{1}{SNR^{t}} \end{array}$$

for the local and remote DPD, respectively. Here, ${\bar{P}}_{ACLR}$ is the ACLR of the input signal, $P_{ACLR}^{t}$ is the ACLR obtained after predistorting the signal (applying coefficients $s_{t}$), and $SNR^{t}$ is the SNR received (analogously). We used the negative as the RL’s objective to maximize the reward. Actually, for the local case, this was the natural choice, but, for the remote case, nothing prevents one using the SNR directly. However, we retained the local case intuition as it yielded superior responses in our experiments.

While the coefficient update mechanism in DRL-DPD may appear similar to that of closed-loop Direct Learning Architectures (DLAs), such as the one presented in [22], there are key differences in methodology and objective. In closed-loop DLA, the coefficient update is derived analytically through gradient-based optimization using the observation path, often assuming a specific error signal model. By contrast, DRL-DPD treats coefficient tuning as an RL problem, where updates are not computed from explicit error feedback but learned through interactions with the environment to maximize a cumulative reward. This distinction allows DRL-DPD to operate under more general conditions, including scenarios with non-differentiable performance metrics or where analytical models of the system are unavailable or unreliable.

## 3. Results on Simulated Environments

In this section, we present the results obtained from experiments conducted in simulated environments to evaluate the performance of the proposed DRL-based DPD framework. We first describe the simulation setup used to model the PA and the training environment for the DRL agent. Subsequently, we analyze the learning behavior of the agent and assess its effectiveness in both local and remote scenarios.

### 3.1. Simulation Setup

To simulate the environment, we simulated the PA using a Saleh model [23], setting a backoff of 10 dB. Specifically, we used a 400 MHz bandwidth 5G-NR OFDM signal consisting of 10 k samples with a carrier frequency of 28 GHz and an MP model with order K=5 and M=1 (no memory). The 10 k sample block corresponded to the capture of a single OFDM symbol. In our 5G FR2 setup (120 kHz subcarrier spacing), one symbol lasted approximately 8.33μs, and our 10μs capture window (assuming a 1 GSa/s sampling rate) was sufficient to contain it. While capturing multiple symbols could improve robustness against noise, our measurement was taken at the PA output where the signal was strong, making a single-symbol measurement adequate for reliable ACLR estimation. This approach provided the DRL agent with accurate feedback without the unnecessary computational burden of processing longer captures.

As the brain of the DPD system, we used DDPG [19], a renowned DRL algorithm tailored for continuous control tasks. While exploring alternatives like TD3 and SAC, DDPG consistently outperformed them, prompting our focus on it. DDPG utilizes an actor–critic architecture with two neural networks: the actor and the critic. The actor determines actions based on states, while the critic evaluates these choices, facilitating actor improvement. We designed DDPG from scratch, in order to easily adapt it to our framework, using Python 3.11 and Tensorflow 2.12 [24]. Specifically, we employed three fully connected hidden layers for both actor and critic, with *H* neurons each (leaving it as hyperparameter). Input and output layer sizes instead depended on the total order of the DPD model, *J*. In our experiments, we had a total of ≈530 k parameters since J=5, H=512 and we considered only odd nonlinearities. Generally speaking, there were $2\left(2\lceil \frac{J}{2}\rceil H\right)+2H^{2}$ parameters to train.

While the total number of trainable parameters may seem large, in practice, this is really modest compared to typical deep learning models and remains easily manageable with modern processors. Importantly, the hyperparameters we used in our experiment were not chosen arbitrarily but were computed via Bayesian optimization using the Optuna framework [25]. The optimized configuration, summarized in Table 1, consistently emerged as the best trade-off between training stability, sample efficiency, and final DPD performance (ACLR/SNR improvement) across repeated trials. Because the primary objective of this work was to validate the feasibility and effectiveness of the DRL-DPD approach, our design intentionally prioritized robustness and convergence; further complexity reduction is nevertheless a natural and practical direction for prospective work (see Section 5).

During development we also evaluated complementary techniques, such as prioritized experience replay buffers and noisy nets [26] (i.e., neural networks incorporating random noise), which improved exploration and generalization and are included in the results shown below.

### 3.2. Results

**Figure 3:**
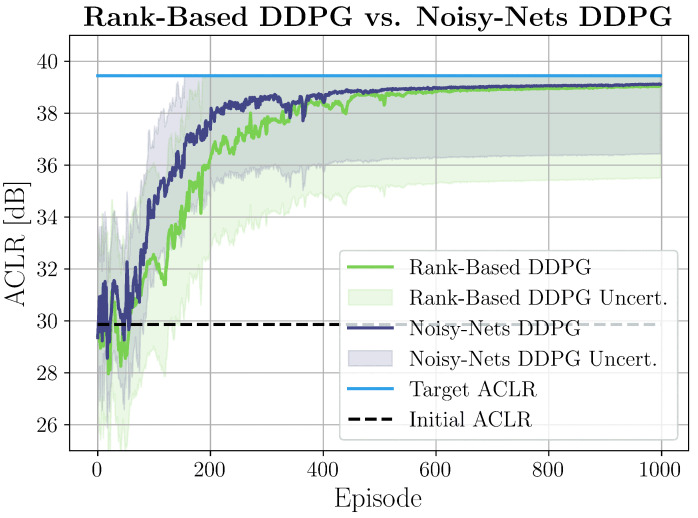
Agent’s learning with related uncertainty in a local setting. Each episode corresponded to 10 complete coefficient update cycles. The azure line corresponds to ${\bar{P}}_{ACLR}$, the dashed black line is the very first ACLR measurement, while the purple and green lines are the ACLR measurements over time of the noisy nets and rank-based DDPG, respectively.

The results for the local scenario are shown in Figure 3. Although DPD operated in a continuous manner, we divided the learning process into discrete episodes to enable structured coefficient updates and systematic performance evaluation. In RL, an episode refers to a full interaction cycle between the agent and the environment: the agent repeatedly selects actions, receives rewards, and transitions through states until a terminal condition is reached or the environment resets. In particular, in our scenario, each episode consisted of exactly 10 sequential steps, where each step represented one complete coefficient update cycle. Within each step, the following operations occurred:

1. Update DPD coefficients based on current state and policy
2. Apply updated coefficients to pre-distort the input signal
3. Transmit the pre-distorted signal through the PA
4. Measure ACLR/SNR and provide feedback to the DRL agent

**Figure 4:**
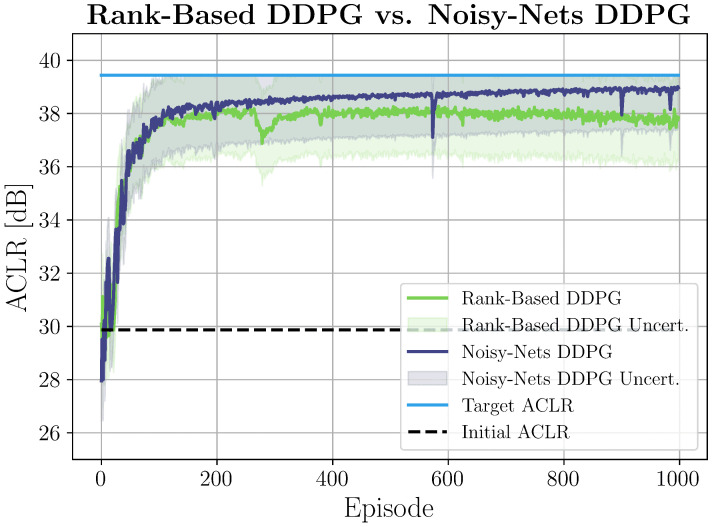
Agent’s learning with related uncertainty in a remote setting. Each episode corresponded to 10 complete coefficient update cycles. The azure line corresponds to ${\bar{P}}_{ACLR}$, the dashed black line is the very first ACLR measurement, while the purple and green lines are the ACLR measurements over time of the noisy nets and rank-based DDPG, respectively.

Denoting as $s_{t}^{i}$ the state at time *t* of the *i*-th episode, we set $s_{0}^{0}=\left[\begin{array}{ccc} 1 & 0 & \ldots 0 \end{array}\right]$, that is, we started by applying no filter. Then, for every episode i>0, we re-initialized the agent to the visited state (previously used coefficients) that returned the highest reward (this information could be easily extracted from the replay buffer). This ensured that the agent started each new episode from the best coefficients found so far. We can clearly see how at the beginning, the agent explored the environment and, after almost 800 updates (i.e., 80 episodes), it started gaining more and more dBs. In particular, here, we report the DDPG techniques that provided the best results. Noisy nets were confirmed to perform better not only in the local case but also in the remote configuration, as shown in Figure 4. In this setup, the agent was demonstrated to be even faster, starting to optimize the SNR after just 150 updates (i.e., 15 episodes). However, remote learning showed slightly increased noise and instability compared to local setups. This was likely due to the fact that the SNR was not a direct measure of PAs’ nonlinearities. Nevertheless, the obtained results show that DRL can effectively be applied to local and remote DPD systems to linearize the PA with low cost and complexity. To have more evidence of its effectiveness, we tested DRL-DPD on hardware for the local configuration.

## 4. Local DRL-DPD on Hardware

In this section, we evaluate the proposed DRL-DPD framework in a real hardware environment to validate its effectiveness beyond simulations. We first describe the hardware testbed and experimental setup used to implement the local DRL-DPD system. Then, we introduce a novel Safe-RL strategy, CRE-DDPG, designed to ensure reliable exploration when operating on physical hardware. Finally, we present the experimental results, analyzing the performance and robustness of the proposed approach.

### 4.1. Experimental Setup

**Figure 5:**
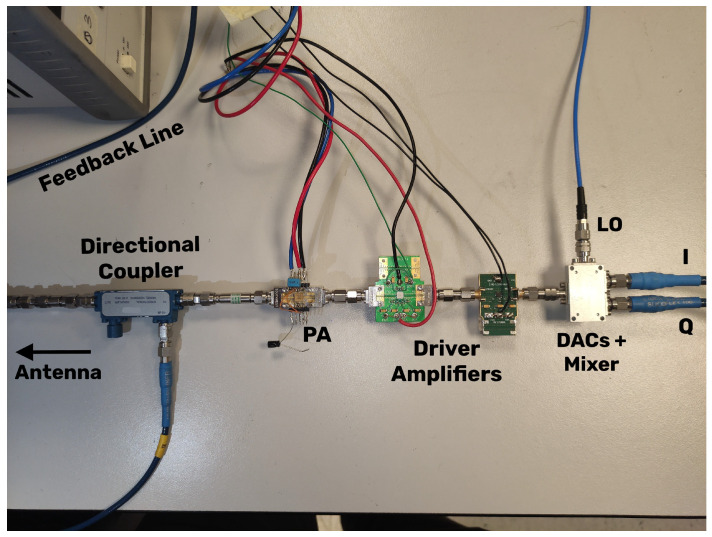
Hardware setup used to test local DRL-DPD in the Huawei Milan Research Center.

Figure 5 depicts the setup we used for testing local DRL-DPD in a real scenario. This architecture mirrors the one shown in Figure 1, but with hardware components. The only difference is that, just for time and resources requirements, we did not use a power detector to measure the ACLR but extracted it from the workstation where the DRL agent was also implemented. Related to this, we can see in the top left of the figure the feedback-line that brought the portion of power provided by the directional coupler to the workstation. Specifically, in this experiment, we utilized a broadband 1W GaAs PA operating from 27 GHz to 32 GHz, with a nominal output power of 27 dBm, a 1-dB compression point (P1dB) of 29 dBm, a saturation output power (Psat) of 30 dBm, and an LO that set the frequency at 27.5 GHz. The signal used mirrored the one used in the local environment, i.e., a 400 MHz bandwidth 5G-NR OFDM signal consisting of 10 k samples with a carrier frequency of 28 GHz and a PAPR of approximately 10 dB. With regard to the DRL environment, nothing changed except the new environment settings and the additional requirements for communicating with the instruments.

### 4.2. CRE-DDPG Safe-RL Exploration

In practical applications, to ensure compliance with regulatory standards and prevent potential damage to the PA, it was crucial to minimize the dispersion of out-of-band power. This necessitated enhancing the agent’s awareness and caution during its exploration of the environment. Consequently, the agent needed to adjust the DPD coefficients at each step without encountering ACLR measurements that fell below a predefined safety threshold. To address this, we developed a specialized Safe-RL algorithm tailored to our needs, based on DDPG (with noisy nets), named CRE-DDPG (Cautious-Recoverable-Exploration DDPG). This algorithm maintains simplicity by not introducing additional complexity beyond the standard DDPG. Essentially, CRE-DDPG modulates the agent’s actions by considering two factors: the received reward and the magnitude of the chosen action. It employs an attenuator, denoted as ρ, to adjust the magnitude of the intended action based on these factors:

$$\rho =1-e^{-\lambda \frac{\Delta h_{t}}{\sqrt{{∥a_{t}∥}_{2}}}}$$

Here, λ>0 is a hyperparameter that dictates the attenuation’s intensity (set to 5 in our configuration), ${∥a_{t}∥}_{2}$ is the L2-norm of the action vector, and $\Delta h_{t}$ signifies the normalized difference between the previously observed reward and the current threshold, defined as $\Delta h_{t}=\left(r_{t-1}-h\right)/h$. The core idea is that when $\Delta h_{t}>0$, the closer the ACLR is to the safety threshold and the more the agent intends to deviate from the current coefficients, the greater the attenuation applied. If $\Delta h_{t}<0$, a recovery strategy is initiated. Practically, during each episode, the actions taken are stored in a buffer A. When the aforementioned condition is met, the agent retraces its steps by executing the reverse of the actions taken, with a slight noise added to ensure that the agent gains further insights into the environment by exploring the vicinity of safe states. Once the agent reaches the vicinity of the initial safe state, it resumes taking small random actions.

**Figure 6:**
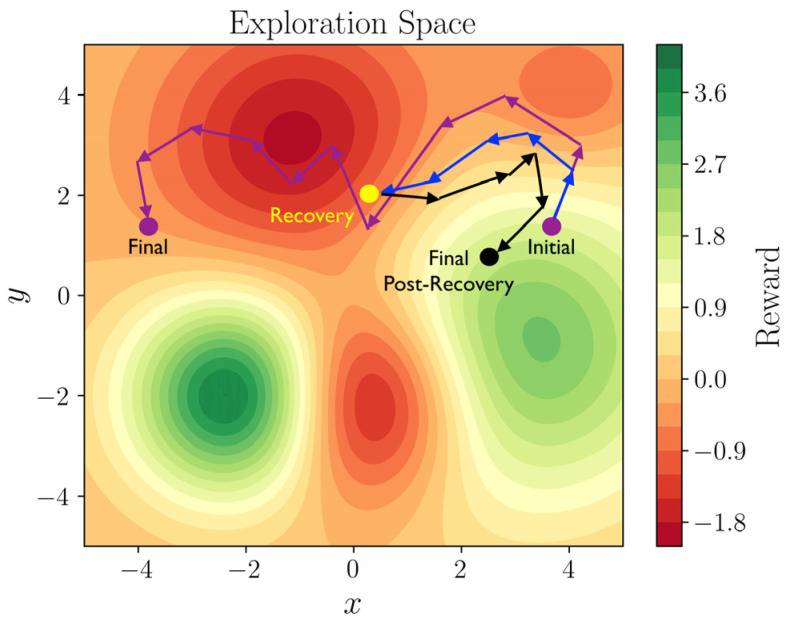
Illustration of CRE-DDPG algorithm in a fictitious space.

Figure 6 provides a visual representation of the algorithm in a fictitious space (the reward scale is irrelevant). In the figure, the actions a conventional DDPG algorithm might take are depicted in purple. As observed, the algorithm may visit unsafe states and subsequently correct itself. In contrast, our algorithm, shown in blue, avoids this by activating the recovery plan as soon as the observed reward surpasses the threshold, guiding the agent back to a safe state near the initial one due to the introduced noise. These corrective actions are shown in black.

**Figure 7:**
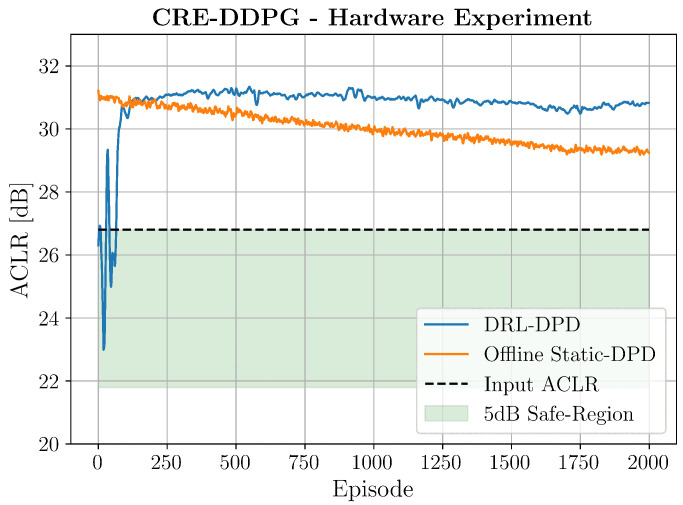
ACLR measurements experienced by CRE-DDPG over time on the hardware setup.

As demonstrated in Figure 7, we established a 5 dB safety margin relative to initial ACLR measurements to prevent threshold violations during exploration. While sufficient for preventing PA damage in our controlled experiments, real-world deployments must adhere to strict regulatory standards. As a matter of fact, 3rd-Generation Partnership Project (3GPP) specifications require base stations to maintain specific ACLR levels for adjacent channels, making compliance essential for licensed operation. The challenge arises during RL training, where exploratory actions may temporarily degrade ACLR below acceptable levels. To address this in practical deployments, we propose maintaining the PA active while configuring the Time Division Duplex (TDD) switch to route signals to the receiver path rather than the antenna. This enables continuous DPD adaptation while preventing over-the-air transmission, ensuring that exploratory actions cannot cause regulatory violations or adjacent channel interference. Algorithm 1 presents the CRE-DDPG pseudocode, with CRE-specific enhancements to DDPG highlighted in blue.
**Algorithm 1** CRE-DDPG 1:Initialize critic network $Q(s,a|\theta^{Q})$ and actor $\mu (s|\theta^{\mu })$ with weights $\theta^{Q}$ and $\theta^{\mu }$ 2:Initialize target network $Q^{\prime }$ and $\mu^{\prime }$ with weights $\theta^{Q^{\prime }}\leftarrow \theta^{Q}$ and $\theta^{\mu^{\prime }}\leftarrow \theta^{\mu }$ 3:Initialize replay buffer R 4:Set safety threshold *h* 5:**for** episode i=1,…,M **do** 6:   Initialize a random process N for action exploration 7:   recovery ← False 8:   Initialize A as empty list 9:   Set new initial state $s_{1}$ and observe reward $\bar{r}$10:   **for** t=1,…,T **do**11:     Compute safety normalized margin $\Delta h_{t}=\frac{\bar{r}-h}{h}$12:     **if** $\Delta h_{t}>0\;\wedge \;\neg$ recovery **then**13:        Select action $a_{t}=\mu \left(s_{t}|\theta^{\mu }\right)+N_{t}$14:        Compute attenuator $\rho =1-e^{-\lambda \frac{\Delta h_{t}}{\sqrt{{∥a_{t}∥}_{2}}}}$15:        Update action $a_{t}\leftarrow \rho a_{t}$16:        Append $a_{t}$ to A17:     **else**18:        recovery ← True19:        **if** A is empty **then**20:          Take small random action around safe state, i.e., $a_{t}=N\left(0,\sigma_{e}\right)$21:        **else**22:          $a_{t}=-A_{t-1}+\mathrm{sign}\left(-A_{t-1}\right)\;U\left(m,M\right)$23:          Remove last element of A, i.e., $A_{t-1}$24:        **end if**25:     **end if**26:     Execute action $a_{t}$ and observe reward $r_{t}$ and new state $s_{t+1}$27:     Set $\bar{r}=r_{t}$28:     Store transition $\left(s_{t},a_{t},r_{t},s_{t+1}\right)$29:     Sample a random minibatch of *N* transitions $(s_{i},a_{i},r_{i},s_{i+1})∼R$30:     Set the target value $y_{i}$ as$$y_{i}=r_{i}+\gamma Q^{\prime }\left(s_{i+1},\mu^{\prime }\left(s_{i+1}|\theta^{\mu^{\prime }}\right)|\theta^{Q^{\prime }}\right)$$31:     Update critic by minimizing the loss: $L=\frac{1}{N}\sum_{i}^{M}{\left(y_{i}-Q\left(s_{i},a_{i}|\theta^{Q}\right)\right)}^{2}$32:     Update actor policy by using the sampled policy gradient:$$\nabla_{\theta^{\mu }}J\approx \frac{1}{N}\sum_{i}\nabla_{a}Q\left(s,a|\theta^{Q}\right){|}_{s=s_{i},a=\mu \left(s_{i}\right)}\nabla_{\theta^{\mu }}\mu \left(s|\theta^{\mu }\right){|}_{s_{i}}$$33:     Update the target networks:
$$\theta^{Q^{\prime }}\leftarrow \tau \theta^{Q}+\left(1-\tau \right)\theta^{Q^{\prime }}$$
$$\theta^{\mu^{\prime }}\leftarrow \tau \theta^{\mu }+\left(1-\tau \right)\theta^{\mu^{\prime }}$$
34:   **end for**35:**end for**

### 4.3. Results

We tested the CRE-DDPG agent for 2000 episodes using the same hyperparameters of the simulated environment and the results are reported in Figure 7. In the figure, the orange line represents the ACLR measured over time by using some coefficients computed before launching DRL (static DPD), which were kept fixed over the entire simulation. This is useful because it clearly shows that the PA’s behavior changed over time, and coefficents that were good before became obsolete then. The blue line, meanwhile, shows the reward experienced by CRE-DDPG over time. In line with the simulation results, the agent started to linearize the PA more and more after ≈850 coefficient updates.

Notably, the DRL agent remained resilient to environmental variations, consistently maintaining performance while reliably operating within the safe region.

**Figure 8:**
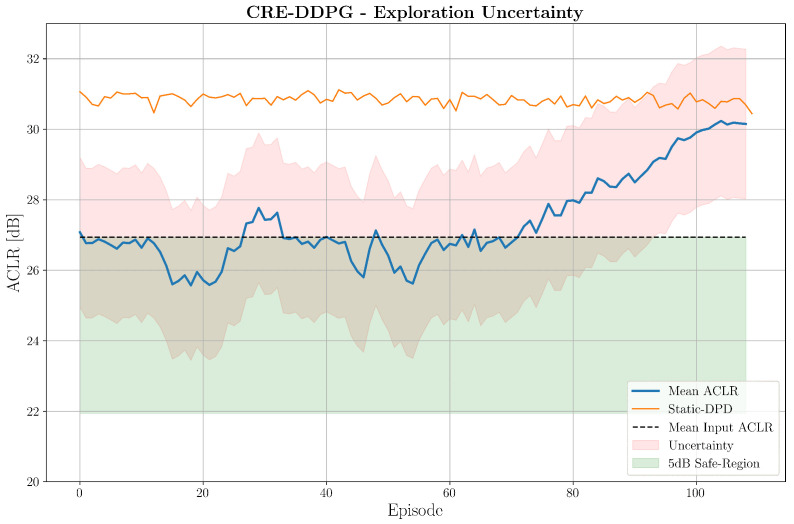
CRE-DDPG reliability assessment over 25 simulations.

In particular, to assess the reliability of CRE-DDPG, we tested it on 25 simulations. In all cases, the agent mantained a safe-behaviour and started to improve the ACLR after no more than 950 updates. This is illustrated in Figure 8, where we can see the mean ACLR measurements over the simulations and the corresponding uncertainty.

## 5. Conclusions

### 5.1. Final Considerations

In this paper, we established a foundation for further advancements. There are several opportunities to enhance the performance and applicability of our approach.

First, additional hardware experiments are necessary to extend our findings. Similar to our work in the simulated environment, future efforts could explore diverse hardware implementations. For example, adding complexities such as memory effects to the DPD model, using alternative models like the Generalized Memory Polynomial, or testing under challenging conditions (e.g., high temperature variations) could provide valuable insights. Given the strength of DRL in handling complex tasks, we remain confident in the potential of DRL-DPD to deliver promising results.

Additionally, future studies could investigate model compression techniques to further optimize the actor and critic architectures. While our current 530k-parameter DDPG model provides good performance, techniques such as pruning or knowledge distillation could potentially reduce the parameter count without significantly compromising DPD effectiveness. Such optimizations could enhance deployment feasibility on resource-constrained embedded platforms and reduce computational overhead in real-time applications.

Improvements to the CRE-DDPG algorithm also offer exciting possibilities. While this study presented a foundational version, enhancements (e.g., replacing random actions with more informed strategies after recovery) could yield better performance. Alternatively, Safe-RL algorithms from the literature could be explored. Moreover, the application of the DRL agent in real-world remote DPD scenarios remains an important area for future research, as time constraints limited us to simulated environments. Evaluating DRL-DPD in practical remote implementations will further validate its effectiveness.

Another potential direction for future work involves exploring alternative hardware architectures that could further optimize implementation efficiency. For instance, an alternative architecture could employ a different feedback approach that eliminates the narrowband filtering stage altogether, compared to what we proposed in Figure 1. Instead of measuring spectral sidebands through our targeted filter and power detector approach, the PA output could be directly compared with a delayed version of the input signal to compute an error signal, e(t), representing the PA’s nonlinearity. This error could then be rectified and its power estimated, providing the DRL agent with alternative feedback for DPD coefficient optimization. Such a design could offer different implementation trade-offs by replacing the selective spectral measurement with a more direct error-based approach and a low-cost ADC. Although this alternative was not validated in the present work, it represents another pathway for DPD implementation while maintaining the core principle of scalar feedback for the DRL agent.

Finally, future studies could also analyze how radio channel propagation conditions, particularly in mobility scenarios, affect the performance of remote DPD estimation. Dynamic environments may make it more difficult to close the DPD loop at the remote receiver while maintaining both SNR and ACLR compliance. These conditions could also increase the number of training episodes required or lead to instabilities, even when using advanced methods such as CRE-DDPG. Addressing these aspects will be essential for ensuring robust operation in practical deployments.

### 5.2. Conclusions

In this paper, we proposed DRL-DPD, a novel DRL-based DPD system designed to reduce computational load, enhance adaptability, and minimize resource use compared to traditional methods for both local and remote DPD setups. Results from simulations and hardware tests unequivocally demonstrate DRL-DPD’s effectiveness in mitigating PA nonlinearities, utilizing a much more cost-effective system. Specifically, our Safe-RL algorithm, CRE-DDPG, equipped the DRL agent with higher awareness during exploration, ensures compliance with PA health and out-of-band power constraints, which is crucial for operation in real deployments.

## Figures and Tables

**Table 1:** OPTUNA hyperparams. γ is the discount factor, *N* the batch size, *H* the number of neurons in the hidden layers, and $\alpha_{A}$ and $\alpha_{C}$ are the actor’s and critic’s learning rate, respectively.

Setup	*N*	$\alpha_{A}$	$\alpha_{C}$	γ	*H*
Local	128	$6\times 10^{-5}$	$7\times 10^{-4}$	0.99	512
Remote	128	$10^{-4}$	$10^{-3}$	0.99	512

## Data Availability

The datasets presented in this article are not readily available due to technical/time limitations. Requests to access the datasets should be directed to damiano.badini@huawei.com.
